# Prevalence, Serotype Distribution and Antimicrobial Resistance of Non-Typhoidal *Salmonella* in Hospitalized Patients in Conghua District of Guangzhou, China

**DOI:** 10.3389/fcimb.2022.805384

**Published:** 2022-02-02

**Authors:** Baiyan Gong, Hong Li, Yulian Feng, Shihan Zeng, Zhenxu Zhuo, Jiajun Luo, Xiankai Chen, Xiaoyan Li

**Affiliations:** ^1^ Fifth Affiliated Hospital, Southern Medical University, Clinical Laboratory, Guangzhou, China; ^2^ Obstetrics Department, Second People’s Hospital of Yibin, Yibin, China; ^3^ KingMed School of Laboratory Medicine of Guangzhou Medical University, Guangzhou, China

**Keywords:** non-typhoidal *Salmonella*, humans, serotype, risk factors, antimicrobial resistance

## Abstract

*Salmonella* infection is a major public health concern worldwide, has contributed to an increased economic burden on the health systems. Non-typhoidal *Salmonella* (NTS) is a common cause of bacterial enteritis in humans, causing 93.8 million cases of gastroenteritis globally each year, with 155,000 deaths. Guangzhou city is situated in the south of China and has a sub-tropical climate, the heat and heavy rainfall helps the spread of NTS. However, no information of NTS infection is available in humans in Conghua District, the largest administrative district of Guangzhou. To understand the prevalence, serotype distribution, risk factors and drug resistance of NTS infection in humans in the survey area, an epidemiological investigation was conducted in hospitalized patients in Conghua District in Guangzhou, China. A total of 255 fecal specimens were collected from hospitalized patients (one each), with a questionnaire for each participant, and NTS infection was identified by culture, as well as serotypes confirmed by slide agglutination tests. An average prevalence of 20.39% (52/255) was observed and three serogroups were identified—serogroup B (n = 46), serogroup C1 (n = 4) and serogroup D1 (n = 2). Among them, *Salmonella* Typhimurium (n = 39) was the most common serotype. Children aged <3 years were observed to have a statistically higher prevalence of NTS infection than adults (25.15% versus 4.65%, P = 0.006); children with artificial feeding had a statistically higher prevalence than those with breastfeeding (30.77% versus 8.33%, P = 0.044). Antimicrobial resistance testing revealed that the majority of strains were resistant to ampicillin (92.16%), as well as 47.06% of all strains were multi-drug resistant. Therefore, it is necessary to continuous monitoring and rational use of antibiotics, which will be helpful to reduce the prevalence of resistant strains. These data will aid in making efficient control strategies to intervene with and prevent occurrence of salmonellosis.

## Introduction

*Salmonella* infection is a major public health concern worldwide, in both industrialized and developing countries, has contributed to an increased economic burden on the health systems. Non-typhoidal *Salmonella* (NTS) is a common cause of bacterial enteritis in humans, causing 93.8 million cases of gastroenteritis globally each year, with 155,000 deaths (Ngogo et al., 2020).

NTS causes a disease characterized mainly by gastroenteritis, a localized infection of the terminal ileum and colon characterized by diarrhea, vomiting and abdominal cramps (Lokken et al., 2016). However, clinical signs and symptoms are variable depending on the health status of infected hosts, and the genetic background and infective doses of this pathogen (Blaser and Newman, 1982; Hu et al., 2021). Usually, asymptomatic infection, diarrhea and other common symptoms of gastroenteritis occurs in immunocompetent or healthy individuals while bacteremia or focal infections at systemic sites may occur in immunodeficient or immuno-compromised individuals, such as infants, the elderly, individuals undergoing cancer chemotherapy and those with underlying diseases (Lokken et al., 2016).

According to the White–Kauffmann–Le Minor scheme, *Salmonella* composed of over 2600 serotypes, where most (approximately 1600 serotypes) of the them belong to the subspecies enterica (Elnekave et al., 2020). Though many serotypes (more than 200) have been identified as capable agents of producing pathology in humans, *Salmonella* Enteritidis (*S.* Enteritidis) and *Salmonella* Typhimurium (*S.* Typhimurium) are the two most common serotypes causing human salmonellosis (Xue et al., 2021; Xu et al., 2021).

*Salmonella* has been found in a variety of hosts, including human and animals (such as reptiles, rodents, birds and amphibian). Therefore, infection can be acquired through close-contact with humans or animals infected with *Salmonella*. (Haeusler and Curtis, 2013). However, most human infections of *Salmonella* are considered to result from ingesting contaminated food or water. *Salmonella* is one of the major cause of foodborne illness worldwide, accounts for 80.3 million foodborne cases per year (Mukherjee et al., 2019). Live poultry, eggs, meat or meat products, and dairy products are recognized as one of the major sources of human salmonellosis (Wu et al., 2015). In fact, the risk of *Salmonella* infection is related to a variety of factors, such as different lifestyle, dietary habits and environmental condition. For people living in low-income countries, poor hygiene and lack of access to safe water and food are major cause of high infection rate of *Salmonella* (Galgallo et al., 2018); for people living in developed countries, the consumption of contaminated fresh fruits and vegetables, and meat are the main reason (Carstens et al., 2019).

Currently, worldwide surveillance data has demonstrated a noticeable increase in antibiotic resistance among NTS (Haeusler and Curtis, 2013). Occurrence of antibiotic-resistant NTS are hampering the use of conventional antibiotics, and associated with an increased risk of treatment failure (Su et al., 2004). Meanwhile, the spread of these resistant strains to other parts of the world could pose a major public health challenge, causing significant morbidity and even mortality. Therefore, it is necessary to understand the situation of *Salmonella* infection and drug-resistance in different areas, it will be helpful to guide the clinical rational use of drugs and curb the development of antibiotic resistance.

In China, since the first report of salmonellosis caused by *Salmonella* in humans in Hong Kong in 1977, this pathogen has been attracting increased attention (Chau and Huang, 1977). Guangzhou city is situated in the south of China and has a sub-tropical climate, the heat and heavy rainfall helps the spread of NTS (Guentchev et al., 2016; Milazzo et al., 2016; Kraay et al., 2020). Some studies have reported occurrence of NTS in Guangzhou. However, no reports about prevalence, serotype distribution and antibiotic susceptibility of NTS infection in humans in Conghua District, the largest administrative district of Guangzhou. Conghua District is a region whose economy is relatively backward, agriculture and tourism are important to its economy. According to the clinical results in recent years, it was shown that the prevalence peak of NTS infection occurred in summer or autumn in this area. Thus, a cross-sectional epidemiological survey of NTS was carried out to understand the status of NTS infection, distribution of various NTS serotypes and drug susceptibility in hospitalized patients in Conghua District by culture, serological experiments and antimicrobial susceptibility testing. Meanwhile, possible risk factors for NTS infection were assessed in the investigated areas. The finding from the present study may assist in setting appropriate measures for the prevention and control of salmonellosis, and to provide reference basis for clinical rational drug use.

## Materials and Methods

### Ethics Statement

The present study was reviewed and approved by the Ethics Committee of the Fifth Affiliated Hospital of Southern Medical University. All study participants were informed about the study objectives and the procedures involved in study participation at enrollment. Prior to collection of fecal specimens, signed informed consents was obtained from each adult individual. Additionally, for minors under 18 years of age, a written consent form was signed by the parent or legal guardian.

### Study Area

Guangzhou is the capital city of Guangdong Province in the southern part of China with 11 regions (aka Districts), and has a population of over 18 million. Guangzhou has a subtropical marine monsoon climate. The annual average temperature is 21.5°C–22.2°C, with the lowest (averaging 9°C–16°C) in January and the highest (averaging 28.7°C) in July. The annual rainfall averages nearly 1800 millimeters. Our study was carried out in Conghua District (population over 0.71 million), which is located on the north-east of Guangzhou, with a total area of 1974.5 square kilometers, as the largest administrative district of Guangzhou (geographical coordinates: 23.22° N–23.56° N latitude, 113.17° E–114.04° E longitude).

### Study Population and Collection of Fecal Specimens

During the period between June and October 2020, a cross-sectional investigation of NTS was carried out in the Fifth Affiliated Hospital of Southern Medical University in Conghua District. The hospital is the premier health care provider for local residents. The study population was patients who were clinically suspected with gastrointestinal disease (or “of NTS infection”). Patients or guardians were given clear sampling guidance before collecting stool or anal swab specimens from patients. All the specimens were delivered to the clinical laboratory department within one hour after collection and stored in a freezer at −4°C prior to experiments.

### Questionnaire

A structured questionnaire was administered to each study participant or their guardians. The questionnaire contained some information on socio-demographic characteristics (gender, age and residence), personal hygiene habits (for example, washing hands before meals), and other possible risk factors (for example, source of infant supplementary food) for NTS infection as well as common clinical symptoms (diarrhea, abdominal pain, nausea, emesis, fever, cough, headache and joint pain). Meanwhile, each questionnaire was linked to one stool or anal swab specimen and used for our analysis in the present study.

### Isolation, Identification and Serotyping of *Salmonella*

Stool or anal swab specimens from patients were transferred into 9 mL Selenite brilliant green enrichment broth (SBG) (Jiangmen Kailin Trading Co., Ltd.). The broth was incubated at 37°C for 24 h. Then, streaked onto xylose lysine deoxycholate agar (XLD) plates (Jiangmen Kailin Trading Co.,Ltd), and secondly selected on Hektoen Enteric agar (HE) plates (Jiangmen Kailin Trading Co., Ltd). These inoculated plates were incubated in 35°C, CO_2_ incubator for 18–24 h. *Salmonella* was identified as transparent colonies with central black point. These suspected colonies were further subjected to biochemical tests by VITEK-Compact 2 automatic bacterial identification system (BioMérieux, Marcy-l’Étoile, France). The serotyping of *Salmonella* isolates was performed by the slide agglutination method using commercial (somatic) and H (flagellar) antigens (Ningbo Tianrun bio-pharmaceutical Co.,Ltd.) according to Kauffman-White scheme.

### Antimicrobial Susceptibility Testing

Antimicrobial susceptibility testing of culture positive bacterial isolates was performed by the standard disk-diffusion method (Kirby-Bauer) as recommended by the Clinical and Laboratory Standards Institute (CLSI). Combined with the clinical medication situation of the study hospital, the following antimicrobial agents (concentration) were used: Azithromycin (15 μg), Ampicillin (10 μg), Ciprofloxacin (5 μg), Chloramphenicol (30 µg), Ceftriaxone (30 µg) Amoxicillin-clavulanic acid (20/10 μg). In addition, the Minimum Inhibitory Concentrations (MICs) were determined using E-tests for seven antibiotics (Sulphamethoxazole/Trimethoprim, Piperacillin/Tazobactam, Cefepime, Ceftazidime, Imipenem, Levofloxacin and Cefoperazone/Sulbactam). The results of antibiotic susceptibility testing (sensitive, intermediate, and resistant) were read and interpreted with reference to the CLSI 2021 criteria. In addition, in the absence of CLSI criteria, *in-vitro* studies have used cefoperazone breakpoints for reporting the susceptibility results for cefoperazone-sulbactam. Thus, resistance was defined as ≥ 64 mg/L with a fixed ratio (2:1) of cefoperazone and sulbactam (the latter was used at 8 mg/L), intermediate susceptibility as 32/16 mg/L and susceptibility as < 16/8 mg/L (Jones et al., 1987).

### Quality Control

Escherichia coli ATCC 25922 was used as a quality control strain in the present study.

### Statistical Analysis

Descriptive statistics were used to determine the relationships between prevalence of NTS and the variables listed in **Supplementary Table 1** in the present study. All statistical analyses were performed with Statistical Package for the Social Sciences (SPSS) 19.0. Pearson chi-square (χ 2) and Fisher’s exact tests were used to determine statistical significance, and *P* value of < 0.05 was considered significant.

## Results

### Prevalence of *Salmonella* Infection

A total of 255 fecal specimens were screened for the presence of *Salmonella* by the methods mentioned above. Fifty-two specimens (20.39%, 52/255) were positive for NTS.

A number of possible risk factors related to NTS infection were analyzed in the present study, including gender, clinical signs, personal eating and hygiene habits and other risk factors (**Supplementary Table 1**). The prevalences of NTS were statistically different in two categories of possible risk factors: children (aged < 3 years) (25.15%, 43/171) versus adults (aged >18 years) (4.65%, 2/43) (*P* = 0.006), and artificial feeding (artificial milk) (30.77%, 40/130) versus breastfeeding (8.33%, 2/24) (*P* = 0.044). In other groups, no statistical significance was found although there were differences in infection rates of NTS (**Supplementary Table 1**).

In the present study, 151 patients had at least one of the eight clinical symptoms (diarrhea, abdominal pain, nausea, emesis, fever, cough, headache, joint pain) listed in **Supplementary Table 2**. Among them, the most common presenting symptom was diarrhea (72.85%, 110/151), followed by fever (52.98%, 80/151) and emesis (23.18%, 35/151) (**Supplementary Table 3**). However, by statistical calculation, no relationship was found between clinical symptoms and NTS infections (*P* > 0.05)

### Serotype Distribution of NTS

For the 52 NTS cases, the dominant serogroup detected in the present study was serogroup B (88.46%, 46/52), followed by serogroup C1 (7.69%, 4/53) and serogroup D1 (3.85%, 2/52). Among them, 39 isolates were further identified as *S.* Typhimurium. In the present study, serogroup B was dominant in all age groups. And this phenomenon was more pronounced in the children aged < 3 years (93.02%, 40/43) (**Supplementary Table 4**).

### Relationship Between Clinical Symptoms and *Salmonella* Serotypes

Among the 52 NTS cases, 37 had at least one of the eight clinical symptoms listed in the present study at the time of sampling, with 27, three and one being found to be infected with serogroup B, serogroup C1 and serogroup D1, respectively. For cases infected with serogroup B including *S.* Typhimurium, diarrhea (58.70%), fever (45.65%) and emesis (15.22%) are the three most common clinical symptoms. For cases infected with serogroup C1 or D1, both diarrhea (75% or 50%) and fever (50% or 50%) are common symptoms (**Supplementary Table 5**).

### Antimicrobial Susceptibility Pattern

Thirteen antimicrobial agents were included in this study, and antimicrobial susceptibility was performed on 51 strains (**Table 1**). All strains were sensitive to imipenem, levofloxacin and cefoperazone/sulbactam. The majority of strains were resistant to ampicillin (92.16%), no strains were found resistant to piperacillin/tazobactam-resistant or amoxicillin-clavulanic acid. All strains showed low prevalence of cephalosporin resistance, and the prevalence rates for resistance to cefepime, ceftriaxone and ceftazidime were 13.73%, 19.61% and 17.65%, respectively. Twenty-four (47.06%) of the 51 strains showed multidrug resistance (MDR, resistant to at least three antimicrobials) (**Table 2**).

**Table 1:** Antimicrobial susceptibility pattern of NTS strains in the present study (N = 51).

Antimicrobials (abbreviations)	Number of resistant strains (%)				
	Susceptible	Intermediate resistance	Resistant	MIC50	MIC90
Azithromycin (AZM)	50 (98.04)	0	1 (1.96)		
Ampicillin (AMP)	4 (7.84)	0	47 (92.16)		
Sulphamethoxazole/trimethoprim (SXT)	33 (64.71)	0	18 (35.29)	1	16
Ciprofloxacin (CIP)	23 (45.10)	26 (50.98)	2 (3.92)		
Chloramphenicol (CHL)	33 (64.71)	0	18 (35.29)		
Piperacillin/tazobactam (TZP)	50 (98.04)	1 (1.96)	0	4	4
Cefepime (FEP)	42 (82.35)	2 (3.92)	7 (13.73)	0.125	16
Ceftriaxone (CRO)	41 (80.39)	0	10 (19.61)		
Ceftazidime (CAZ)	42 (82.35)	0	9 (17.65)	0.25	32
Amoxicillin-clavulanic acid (AMC)	46 (90.20)	5 (9.80)	0		
Imipenem (IPM)	51 (100.00)	0	0	0.25	0.25
Levofloxacin (LVX)	51 (100.00)	0	0	1	1
Cefoperazone/sulbactam (CSL)^a^	51 (100.00)	0	0	8	16

a Criteria as published by the CLSI for cefoperazone used for cefoperazone-sulbactam.

**Table 2:** Distribution of NTS strains resistant to antibiotics and resistant spectrum.

Resistant types	No. strains (%)	Resistant spectrum (n)
0^a^	4	
1	18	AMP (18)
2	5	AMP-CHL (2); AMP-SXT (3)
≥3	24	AMP-SXT-CHL (13); AMP-CAZ-CRO-FEP (2); AMP-CRO-CHL-CIP (1); AMP-SXT-CHL-CIP (1); AMP-CAZ-CRO-FEP (5); AMP-CAZ-CRO-FEP-SXT (1); AMP-CAZ-CRO-FEP-AZM-CHL (1)

a 0 indicates no resistant.

## Discussion

NTS has been reported in humans worldwide. In the present study, 20.39% (52/255) of hospitalized patients of a large hospital in the north-east of Guangzhou City were confirmed to be infected with NTS. The present prevalence was higher than that of the previous reports in patients with gastrointestinal complaints of 13.80% (41/297) in Tanzania (Ngogo et al., 2020) and 6.2% (59/957) in Ethiopia (Eguale et al., 2015), as well as in pet caregivers of 17.86% (25/140) in Thailand (Wu et al., 2020). The variation between studies on *Salmonella* infection could be due to multiple factors, such as differences of enrolled patient characteristics (gender, age and residence), time of sample collection (seasonal variation), and possible risk factors (Ngogo et al., 2020).

In the present study, a statistical difference in the prevalence of NTS was observed between children (25.15%, 43/171) and adults (4.65%, 2/43). Children have been identified as a population group at risk for *Salmonella*i infection because of immature immune system and lack of good hygienic habits (Mascaro et al., 2017). There have been some studies reporting high prevalence of *Salmonella* in children, such as 10.3% (33/320) in Iraq (Harb et al., 2017), and 9.7% (258/2658) in China (Liu et al., 2021). In addition, this result may have been affected by selection bias (detection bias). The majority of NTS infections in humans are self-limiting, some patients can heal themselves without treatment. However, there is a greater proportion of symptomatic infections among children, who also are more likely to seek medical help and therefore to have a stool examination for the detection of *Salmonella* (Mascaro et al., 2017).

The present high prevalence may also be related to the sampling period from June to October, the average temperature is above 24°C. This result was similar to that of a report conducted by the Center for Disease Control and Prevention (CDC), which found that most *Salmonella* infections occur between June and October in the U.S. (Centers for Disease Control and Prevention (2013)). Previous studies have shown that higher temperatures can promote the growth and reproduction of *Salmonella*, as well as transmission of salmonellosis (Guentchev et al., 2016). In addition, warmer weather may increase the frequency of eating out, and lead to an increased risk of *Salmonella* infection (Milazzo et al., 2016).

In our analysis of multiple possible risk factors for *Salmonella* infection, artificial fed (non-breast-fed) infants have a higher probability of *Salmonella* infection than do breast-fed infants (30.77% versus 8.33%). Powdered infant formula (PIF) is not a sterile product and may be intrinsically contaminated with *Salmonella*. It is reported that more than ten outbreaks of salmonellosis conducted in infants and were linked to the consumption of PIF (Jones et al., 2006). Unlike artificial feeding, breastfeeding is most likely protective for babies because of passes on helpful maternal immunities. Numerous studies have shown that breastfeeding reduces the risk of a number of diseases in infants, including salmonellosis (Jones et al., 2006; Ehlayel et al., 2009).

In the present study, three serogroups (serogroup B, serogroup C1 and serogroup D1) were identified, and serogroup B was the most common (88.46%, 46/52) in humans in the investigated areas. And the most frequently isolated serotype is *S.* Typhimurium (n = 39), which was similar to findings previously reported in some studies in Guangdong Province in China (Zhang et al., 2016; Zhang, 2018; Huang, 2020). In fact, epidemiological data have revealed that the distribution of NTS serotype in humans is different among geographic areas. As reported by Li Y, *S.* Enteritidis is the dominant serotype in north China, in contrast, *S.* Typhimurium is responsible for more infections than *S.* Enteritidis in south China (Li et al., 2018).

Though serogroup B was dominant in all age groups, children aged < 3 years were more likely to be infected by serogroup B, especially *S.* Typhimurium. This might be related to children’s mobility activities, such as crawling, touching or biting toys/food dropped and picked up from floor, increasing the opportunity of transmission of *Salmonella* infection from households flooring materials contaminated with *Salmonella* to humans. As a previous study showed that, over 99% of *S.* Typhimurium were transferred from tile to food after five seconds of exposure to tile (Dawson et al., 2007).

NTS has been reported to give rise to clinical symptoms, like diarrhea, abdominal pain, nausea, emesis, fever, cough, headache, joint pain. In the present study, no associations were observed between NTS infection and clinical symptoms as well as each symptom category. In fact, besides immune status of individuals, the occurrence and the severity of clinical symptoms are also observed to be related to the infection intensity and the duration of excretion of NTS, as well as the virulence of serotypes (Haeusler and Curtis, 2013). In addition, clinical symptoms might be caused by other factors, even changes in diet can affect fecal consistency (e.g., the presence or absence of diarrhea).

With the extensive use of the antibiotic, especially abuse, bacterial resistance has been recognized as a worldwide health problem. Ampicillin, chloramphenicol, and sulphamethoxazole/trimethoprim were regarded as first-line antimicrobials used for the treatment of severe salmonellosis for a long time (Andoh et al., 2017). In the present study, resistance to ampicillin, chloramphenicol, and sulphamethoxazole/trimethoprim were 92.16%, 35.29%, and 35.29% respectively. Similarly, some studies in developing countries have found that these first-line antimicrobials have become less effective due to development of resistance (Andoh et al., 2017; Liang et al., 2019; Shen et al., 2020). In the present study, low level of resistance towards fluoroquinolones (3.92% for ciprofloxacin and 0 for levofloxacin) were observed, which was inconsistent with the findings previously reported in some studies in Uganda and Japan (Kobayashi et al., 2014; Ota et al., 2016). This may be due to the fact that 90.38% (47/52) NTS strains isolated from children aged ≤ 5 years in this study, fluoroquinolones were rarely used in children due to articular toxicity (Ke et al., 2020). Similarly, in a study conducted in China, the prevalence of resistance to ciprofloxacin among NTS isolates was 8.05% (147/1826), and children aged <5 years accounted for 73% (1329/1826) of the overall NTS infections (Liang et al., 2015).

Twenty-four strains were resistant to more than three antimicrobial agent, and they were regarded as MDR strains. A related study suggest that hospitalization with bloodstream infection has been reported to be more common in patients infected with antimicrobial-resistant NTS than in patients with pansusceptible infection (Varma et al., 2005). Edward Cox (director of FDA’s Office of Antimicrobial Products in the Center for Drug Evaluation and Research) said that any use of antibiotics, even appropriate therapeutic use, can promote the development of resistant bacteria (http://www.fda.gov/ForConsumers/ConsumerUpdates/ucm378100.htm). Therefore, it is necessary to avoid overusing of antibiotics and.to reduce the emergence of antibiotic-resistant bacterial strains.

This study provides some important insights into the epidemiological characteristics and antibiotic resistance patterns of *Salmonella* infections in this part (Conghua District) of Guangzhou. Bacterial isolation and culture were carried out on the specimens, and the bacteria were identified by VITEK-Compact 2 automatic bacterial identification system. This method has a high identification accuracy and low price, and is a suitable method in hospital microbiological laboratory for testing *Salmonella*. However, there are several limitations of the study. We did not use molecular tools (e.g., whole-genome sequencing) for the detection of *Salmonella*, nor did we type the antimicrobial resistance genes that conferred resistance to the studied antimicrobials. This information is considered essential to study the epidemiology, spread, source attribution of NTS as well as identify mono/polyclonal outbreaks in nosocomial settings. Further studies should focus on genotyping and phylogenetic analysis (e.g., MLST) of *Salmonella*, and typing the antimicrobial resistance genes. These data will be helpful to understand its biology, transmission dynamics and infection sources, as well as develop efficient control strategies to intervene with and prevent occurrence of *Salmonella* infection.

## Conclusion

The present study provided the first report on occurrence and antimicrobial susceptibility of NTS in hospitalized patients in Conghua District in Guangzhou, China. The prevalence of NTS was 20.39% (52/255) in hospitalized patients in the investigated areas, and three serogroups were identified, with serogroup B being dominant (88.46%, 46/52). Among them, *S.* Typhimurium was the most common serotype, representing 84.78% (39/46) of serogroup B strains. Children aged <3 years and those with artificial feeding are more likely to have NTS infection. The highest rate of resistance was recorded in relation to ampicillin (92.16%), chloramphenicol (35.29%) and sulphamethoxazole/trimethoprim (35.29%). High percentage of MDR strains (47.06%, 24/51) emphasizes the importance of the prudent use of antibiotics and antimicrobial resistance monitoring in this district. These data obtained in the present study increase our understanding of the prevalence, serotype distribution, risk factors and drug resistance of NTS infection, and will help develop efficient control strategies to intervene with and prevent the occurrence of salmonellosis in the investigated areas.

## Data Availability Statement

The original contributions presented in the study are included in the article/**Supplementary Material**. Further inquiries can be directed to the corresponding author.

## Ethics Statement

The studies involving human participants were reviewed and approved by the Ethics Committee of the Fifth Affiliated Hospital of Southern Medical University. Written informed consent to participate in this study was provided by the participants’ legal guardian/next of kin.

## Author Contributions

XL and BG conceived and designed experiments. YF and ZZ collected samples. HL and YF performed laboratory experiments. BG, SZ, and JL analyzed and interpreted data. YF, SZ, ZZ, and XC contributed reagents and materials. BG and HL wrote the manuscript. XL revised the manuscript. All authors have read and approved the final version of the manuscript.

## Funding

This project was funded by the Youth Foundation of the National Natural Science Foundation of China (81902104), and the Medical Science and Technology Research Project of Foushan Science and Technology Innovation Program (1920001000717).

## Conflict of Interest

The authors declare that the research was conducted in the absence of any commercial or financial relationships that could be construed as a potential conflict of interest.

## Publisher’s Note

All claims expressed in this article are solely those of the authors and do not necessarily represent those of their affiliated organizations, or those of the publisher, the editors and the reviewers. Any product that may be evaluated in this article, or claim that may be made by its manufacturer, is not guaranteed or endorsed by the publisher.
