# Supplementary material for: Prevalence, Serotype Distribution and Antimicrobial Resistance of Non-Typhoidal Salmonella in Hospitalized Patients in Conghua District of Guangzhou, China
Source: Front Cell Infect Microbiol. 2022 Feb 2;12:805384. doi: 10.3389/fcimb.2022.805384 (PMC8847451; doi:10.3389/fcimb.2022.805384)
Supplement: Supplementary file 1 [file DataSheet_1.docx]

Supplementary Table 1. Assessment of possible risk factors for NTS infection.

| **Variable** | | **Examined no.** | **Positive no. (%)** | **OR (95% CI)^a^** | **χ^2^/p value** |
| --- | --- | --- | --- | --- | --- |
| Gender | Male | 147 | 31 (21.09) | 1.107 (0.596, 2.058) | 0.104/ 0.747 |
|  | Female | 108 | 21 (19.44) |  |  |
| Age (years) | 0–2 | 171 | 43 (25.15) | ref |  |
|  | 3–5 | 25 | 4 (16.00) | 1.764 (0.573, 5.425) | 0.562/ 0.453 |
|  | 6–12 | 16 | 3 (18.75) | 1.456 (0.396, 5.352) | 0.070/ 0.791 |
|  | 13–17 | 0 | 0 (0) | - | - |
|  | >18 | 43 | 2 (4.65) | 6.887 (1.598, 29.676) | **7.501/ 0.006^b^** |
| Residence | Urban | 86 | 20 (23.26) | 1.297 (0.690, 2.439) | 0.656/ 0.418 |
|  | Village | 169 | 32 (18.93) |  |  |
| Drinking boiled water | Yes | 251 | 50 (19.92) | 0.249 (0.034, 1.809) | 0.733/ 0.392 |
|  | No | 4 | 2 (50.00) |  |  |
| Infant and child feeding | Breastfeeding | 24 | 2 (8.33) | ref |  |
|  | Artificial feeding | 130 | 40 (30.77) | 0.205 (0.046, 0.912) | **4.073/ 0.044^b^** |
|  | Both | 25 | 2 (8.00) | 1.045 (0.135, 8.083) | 0/ 1.000 |
| Type of powdered milk | Domestic | 77 | 19 (24.68) | 1.011 (0.502, 2.036) | 0.001/ 0.975 |
|  | Imported | 94 | 23 (24.47) | ref |  |
|  | Both | 8 | 2 (25.00) | 0.983 (0.183, 5.284) | 0/ 1.000 |
| Source of infant complementary food | Homemade food | 137 | 43 (31.39) | ref |  |
|  | Cooked food in the supermarket | 6 | 1 (16.67) | 2.287 (0.259, 20.176) | 0.098/ 0.754 |
|  | Both | 8 | 0 (0) | 0.686 (0.613, 0.768) | -/ 0.105 |
| Preservation of complementary food | Refrigerated | 1 | 0 (0) | 1.386 (1.251, 1.536) | -/ 1.000 |
|  | Room temperature | 10 | 5 (50.00) | 2.590 (0.710, 9.441) | 2.208/ 0.137 |
|  | Cook for immediate consumption | 140 | 39 (27.86) | ref |  |
| Separate chopping boards for handling raw and cooked food | Yes | 35 | 2 (5.71) | 0.436 (0.079, 2.404) | 0.332/ 0.565 |
|  | No | 41 | 5 (12.20) |  |  |
| Washing hands before meals | Yes | 228 | 48 (21.05) | 1.533 (0.506, 4.646) | 0.258/ 0.611 |
|  | No | 27 | 4 (14.81) |  |  |
| Washing hands after using toilets | Yes | 224 | 47 (20.98) | 1.381 (0.503, 3.790) | 0.395/ 0.530 |
|  | No | 31 | 5 (16.13) |  |  |
| Nutritional status | Good | 180 | 37 (20.56) | 0.776 (0.150, 4.004) | 0/ 1.000 |
|  | Fair | 67 | 13 (19.40) | 0.722 (0.130, 3.997) | 0/ 1.000 |
|  | Poor | 8 | 2 (25.00) | ref |  |

*^a^CI, confidence interval.*

*^b^Bold type for values indicates statistical significance.*

Supplementary Table 2. Relationship between NTS infection and clinical symptoms.

| **Variable** | | **Examined no.** | **Positive no. (%)** | **OR (95% CI)^b^** | **χ^2^/p value** |
| --- | --- | --- | --- | --- | --- |
| Clinical symptoms^a^ | Yes | 151 | 37 (24.50) | 1.926 (0.994, 3.729) | 3.855/ 0.050 |
|  | No | 104 | 15 (14.42) |  |  |
| Diarrhea | Yes | 110 | 31 (28.18) | 2.289 (0.876, 5.981) | 2.963/ 0.085 |
|  | No | 41 | 6 (14.63) |  |  |
| Abdominal pain | Yes | 22 | 4 (18.18) | 0.646 (0.204, 2.048) | 0.228/ 0.633 |
|  | No | 129 | 33 (25.58) |  |  |
| Nausea | Yes | 6 | 1 (16.67) | 0.606 (0.068, 5.356) | 0/ 1.000 |
|  | No | 145 | 36 (24.83) |  |  |
| Emesis | Yes | 35 | 7 (20.00) | 0.717 (0.284, 1.810) | 0.499/ 0.480 |
|  | No | 116 | 30 (25.86) |  |  |
| Fever | Yes | 80 | 24 (30.00) | 1.912 (0.887, 4.123) | 2.779/ 0.096 |
|  | No | 71 | 13 (18.31) |  |  |
| Cough | Yes | 19 | 1 (5.26) | 0.148 (0.019, 1.151) | 3.241/0.072 |
|  | No | 132 | 36 (27.27) |  |  |
| Headache | Yes | 8 | 1 (12.50) | 0.425 (0.051, 3.570) | 0.151/0.697 |
|  | No | 143 | 36 (25.17) |  |  |
| Joint pain | Yes | 1 | 0 (0) | 1.327 (1.211, 1.455) | -/ 1.000 |
|  | No | 150 | 37 (24.67) |  |  |

*^a^Indicating people who had at least one of the eight symptoms listed in the present study.*

*^b^CI, confidence interval.*

Supplementary Table 3. Proportion of participants with clinical symptoms.

| **Clinical symptoms** | **All participants** | | ***Salmonella* cases** | |
| --- | --- | --- | --- | --- |
|  | **Frequency (n)** | **Percentage (%)** | **Frequency (n)** | **Percentage (%)** |
| Diarrhea | 110 | 72.85 | 31 | 83.78 |
| Abdominal pain | 22 | 14.57 | 4 | 10.81 |
| Nausea | 6 | 3.97 | 1 | 2.70 |
| Emesis | 35 | 23.18 | 7 | 18.92 |
| Fever | 80 | 52.98 | 24 | 64.86 |
| Cough | 19 | 12.58 | 1 | 2.70 |
| Headache | 8 | 5.30 | 1 | 2.70 |
| Joint pain | 1 | 0.66 | 0 | 0 |
| Total^a^ | 151 |  | 37 |  |

*^a^Indicating people who had at least one of the eight symptoms listed in the present study.*

Supplementary Table 4. Prevalence and distribution of NTS serotype in humans by age.

| **Age group** | **Examined no.** | **Positive no. (%)** | **Serotype (n)** |
| --- | --- | --- | --- |
| 0–2 | 171 | 43 (25.15) | *S.* Typhimurium (34); Serogroup B^a^ (6); Serogroup C1 (3) |
| 3–5 | 25 | 4 (16.00) | *S.* Typhimurium (3); Serogroup D1 (1) |
| 6–12 | 16 | 3 (18.75) | Serogroup B^a^ (1); Serogroup C1 (1); Serogroup D1 (1) |
| 13–17 | 0 | 0 (0) |  |
| >18 | 43 | 2 (4.65) | *S.* Typhimurium (2) |

*^a^Serogroup B excluding S.* Typhimurium

Supplementary Table 5. Relationship between NTS cases and clinical symptoms.

| **Serotype** | **No. Positive /No. examined (%)** | | | | | | | |
| --- | --- | --- | --- | --- | --- | --- | --- | --- |
|  | **Diarrhea** | **Abdominal pain** | **Nausea** | **Emesis** | **Fever** | **Cough** | **Headache** | **Joint pain** |
| Serogroup B | 27/46 (58.70) | 3/46 (6.52) | 1/46 (2.17) | 7/46 (15.22) | 21/46 (45.65) | 1/46 (2.17) | 0 | 0 |
| *S.* Typhimurium | 26/39 (66.67) | 3/39 (7.69) | 1/39 (2.56) | 7/39 (17.95) | 18/39 (46.15) | 1/39 (2.56) | 0 | 0 |
| Other | 1/7 (14.29) | 0 | 0 | 0 | 3/7 (42.86) | 0 | 0 | 0 |
| Serogroup C1 | 3/4 (75.00) | 1/4 (25.00) | 0 | 0 | 2/4 (50.00) | 0 | 1/4 (25.00) | 0 |
| Serogroup D1 | 1/2 (50.00) | 0 | 0 | 0 | 1/2 (50.00) | 0 | 0 | 0 |
| Total | 31 | 4 | 1 | 7 | 24 | 1 | 1 | 0 |
